# Cocoa administration may accelerate orthodontic tooth movement by inducing osteoclastogenesis in rats

**DOI:** 10.22038/ijbms.2018.32967.7881

**Published:** 2019-02

**Authors:** Ananto Ali Alhasyimi, Niswati Fathmah Rosyida

**Affiliations:** 1 Department of Orthodontics, Faculty of Dentistry, Universitas Gadjah Mada, Yogyakarta 55281, Indonesia

**Keywords:** Caffeine, Cocoa, OPG, Orthodontic tooth- movement, RANKL

## Abstract

**Objective(s):**

To investigate the effect of cocoa on orthodontic tooth movement (OTM) rate, osteoprotegerin (OPG), and receptor activator of nuclear factor κ β ligand (RANKL) levels after OTM.

**Materials and Methods:**

A total of 24 Sprague-Dawley rats were included in the study. They were equally divided into two groups: cocoa and control. The upper incisors of all rats were subjected to 35 cN orthodontic force and moved distally with a stainless steel 3-spin coil spring. During OTM, the cocoa group was given 4.8 g of unsweetened cocoa once a day. At 4 subsequent time points (0, 1, 7, and 14 days), the OTM rate was determined by measuring the distance between the mesial tips using a digital caliper, while OPG and RANKL levels were examined based on their gingival crevicular fluid through specific enzyme-linked immunosorbent assay (ELISA). Data gathered were analyzed through independent t-test (*P<*0.05).

**Results:**

The OTM rate of the cocoa group was significantly higher than that of the control group on days 1, 7, and 14 (*P<*0.05). ELISA analysis revealed that the OPG level was significantly lower on day 14. Furthermore, the RANKL level was significantly higher on days 0, 1, and 7 for the cocoa group compared with the control group (*P<*0.05).

**Conclusion:**

These results indicate that cocoa has the potential effect to modulate the OTM rate by inducing osteoclastogenesis, which suppresses the OPG level and stimulates the RANKL level, in rats.

## Introduction

 Orthodontic treatment is a complex treatment with a long duration ranging between 25–35 months for extraction cases, and 21–27 months for non-extraction cases, respectively (1). Acceleration of orthodontic tooth movement (OTM) is advantageous not only to orthodontists because it shortens the duration of treatment, which is correlated with a lower risk of enamel decalcification, caries, destruction of periodontal tissue (as in the case of gingivitis and periodontitis), and root resorption incidence but also to patients because the reduced treatment time results in cost-effective and efficient treatments (2). An effort to increase the rate of tooth movement by promoting osteoclastogenesis has been well-established (3). A prior study demonstrated that transfer of receptor activator of nuclear factor- κB ligand (RANKL) gene to the periodontal tissue has the potential to accelerate OTM by activating osteoclastogenesis. Enhancement of osteoclastogenesis on the pressure side of orthodontic tooth movement results in accelerated orthodontic tooth movement (4). Osteoclastogenesis plays an important role in OTM. The communication between receptor activator of nuclear factor-κB (RANK) expressed on osteoclast precursors and RANKL expressed by osteoblasts is essential for osteoclast formation and activation (5). Osteoblasts also express osteoprotegerin (OPG), which inhibits RANK for RANKL binding, thereby preventing osteoclast differentiation, accelerating osteoclast apoptosis, and finally decreasing osteoclast activity (6). 

In an attempt to accelerate OTM, several novel modalities have been developed, including corticotomy and gene therapy (7), pulsed electromagnetic therapy, mechanical vibration, and low-level laser therapy using light-emitting diodes (8). The use of cyclosporine (9), pantoprazole (10), and *Salvia miltiorrhiza* (11) have been reported to induce accelerated OTM. However, adverse effects and complications in clinical operation have discouraged their extensive application. In recent years, natural materials have been used, developed, and produced massively for medical use (12). Cocoa is a natural material that is being consumed by people worldwide. The health benefits of cocoa have attracted significant attention from scientists. Interestingly, cocoa contains methylxanthine, an active compound that contains a large amount of caffeine (13). The use of caffeine to enhance OTM has been well-documented. Daily ingestion of caffeine in coffee may contribute to the acceleration of tooth movement. Caffeine interrupts the Ca^2+^ ion balance, thereby leading to low bone density, and accelerates bone remodeling, thereby shortening orthodontic treatment duration (14, 15). Furthermore, previous research has shown that traditional Chinese medicine, which contains caffeine, increased the speed of OTM (16). Rat models have been used to adapt the OTM model, and the results of previous research have been generalized for comparison with human subjects. Therefore, this study was intended to investigate the potency of cocoa administration to accelerate OTM by inducing osteoclastogenesis in rat models. 

## Materials and Methods

***Determination of methylxanthines in cocoa using thin-layer chromatography (TLC)***

**Figure 1:**
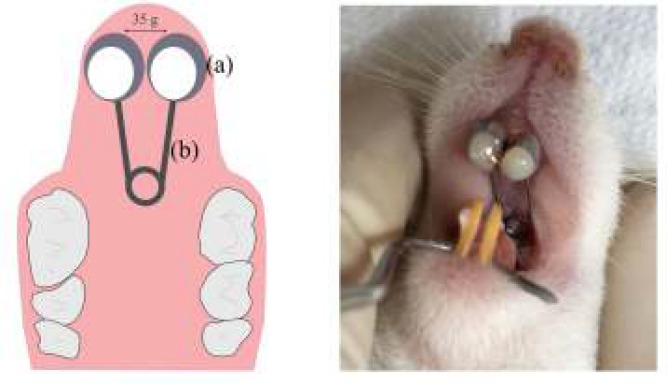
FTIR spectra of the cocoa sample. There are peak wave numbers confirmed as caffein spectra (arrowhead)

Confirmation tests on functional groups that were conducted using Fourier transform infrared (FTIR) in cocoa powder samples demonstrated that caffeine (methylxanthines) was successfully identified from cocoa samples. The presence of caffeine was indicated by strong adsorption peaks at 3335.48 and 2924.02 cm^-1^ (Figure 1). Methylxanthines were also detected from cocoa under UV light at 254 nm using TLC analysis.

***Animal experiments***

Ethical approval was obtained from the Research Ethics Committee of the Integrated Laboratory of Research and Testing, Universitas Gadjah Mada, Indonesia (clearance number 00019/04/LPPT/III/2018). A total of 24 10-week-old male Sprague-Dawley rats (weighing 250–300 g) were enrolled. The animals were housed under normal laboratory conditions and adapted to a 12/12 hr light/dark cycle at 25 ^°^C with humidity range of 64 to 80%. During experiments, the animals were fed standard laboratory pellets and given tap water *ad libitum*.

**Figure 2:**
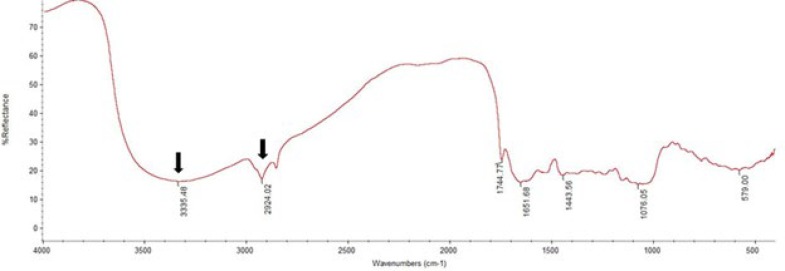
Design of experimental orthodontic tooth movement model in a rabbit model: (a) matrix band and (b) stainless steel wire

**Figure 3:**
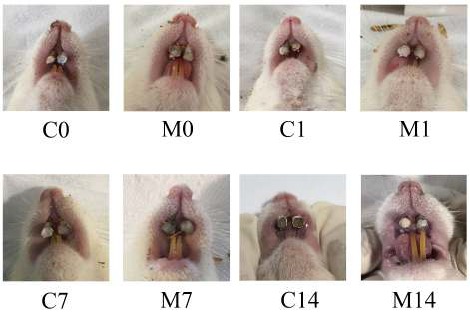
Interincisivus distances (orthodontic tooth movement rates of 2 groups tested; C indicates control group and M indicates cocoa (methylxanthine) group; 0, 1, 7, and 14 indicate the day of examination (0, 1, 7, and 14 days after orthodontic appliance installation, respectively). Orthodontic tooth movement was accelerated in the experimental group by administration of cocoa

**Table 1:** Comparison of orthodontic tooth movement rates (mm) and osteoprotegerin and receptor activator of nuclear factor κ β ligand levels (pg/ml) at each observation time point between 2 groups tested^a^

	**Control Group**	**Cocoa Group**	***P-*** **value**
**OTM rates (mm)**			
Day 0	0.423 ± 0.133	0.401 ± 0.075	0.805
Day 1	0.473 ± 0.101	0.792 ± 0.141	0.033*
Day 7	0.814 ± 0.164	1.103 ± 0.316	0.049*
Day 14	1.127 ± 0.219	1.317 ± 0.149	0.046*
			
**OPG levels (pg/ml)**			
Day 0	0.144 ± 0.058	0.115 ± 0.011	0.486
Day 1	0.168 ± 0.069	0.113 ± 0.017	0.300
Day 7	0.149 ± 0.051	0.125 ± 0.013	0.488
Day 14	0.327 ± 0.095	0.116 ± 0.009	0.018*
			
**RANKL levels (pg/ml)**			
Day 0	0.196 ± 0.063	0.259 ± 0.067	0.041*
Day 1	0.155 ± 0.043	0.355 ± 0.052	0.001*
Day 7	0.391 ± 0.058	0.611 ± 0.104	0.003*
Day 14	0.407 ± 0.056	0.424 ± 0.102	0.812

Values are presented as mean ± standard deviation

a Tested by independent *t*-test of variance

* *P*<0.05, significant differences between groups

The animals were grouped randomly into control and treatment groups, each with 12 animals that were then randomly divided into 4 subgroups of 3 animals corresponding to 4 observation periods 0 (3 hr), 1, 7, and 14 days, after orthodontic appliance installation. During orthodontic appliance installation, the rats were anesthetized by an intramuscular injection of ketamine hydrochloride (Kepro, Netherlands), and xylazine (Xyla, Netherlands) at doses of 35 mg/kg and 5 mg/kg body weight, respectively. In all rats in the two groups, a 3-spin loop spring (2 mm in coil diameter, with arms 6 mm in length, and soldered to the orthodontic band) made of 0.012-inch stainless steel alloy wire (American Orthodontics, USA) was cemented to the upper incisors using glass ionomer luting cement (Fuji I, GC, USA) to move the teeth distally (Figure 2). This method delivered a continuous orthodontic force of approximately 35 g, which was measured using a dynamometer tension gauge (MedKraft Orthodontics, USA). No reactivation of the appliance was performed during the experiment. Immediately after orthodontic appliance installation, 4.8 g of cocoa-containing 2.7 mg of methylxanthine were orally administered to the treatment group once a day using an oral sonde. 

***Measurement of tooth movement rate***

Tooth movement was measured at each time point (0, 1, 7, and 14 days after orthodontic appliance installation). The opening distance between the inner incisal edges of banded-upper incisors represented the OTM rates and was measured using a digital caliper (ProMax, China) with a minimum measurable distance of 0.01 mm. The distance was measured twice, i.e., immediately after installation and on the day of measurement. The difference scores were determined as OTM rates. All measurements were taken by one observer, and each measurement was repeated thrice. The mean of these measurements was used as the representative value for each distance.

***Gingival crevicular fluid (GCF) collection***

Prior to GCF sample collection, the crevicular sulcus of each rat was gently drained by air jets and isolated using cotton rolls to exclude the remaining saliva. GCF of each sample in each group was taken 4 subsequent times (0, 1, 7, and 14), and collected using the smallest diameter of paper point (size #15, Sendoline, UK). The paper point was gently inserted into the gingival sulcus of the mesial side of the teeth (pressure side) and was left in situ for 30 sec. A total of four dipped paper points were then placed into a 1.5 ml Eppendorf tube containing 350 μl of physiological saline solution. Thereafter, the tube was centrifuged for 5 min at 2000 rpm, 4 ^°^C using a microcentrifuge refrigerator (Eppendorf 5424R, USA) to completely elute all the GCF components from the paper points. The paper points were then removed, and the supernatants were kept frozen and stored at −80 ^°^C until assayed (5, 6).

***Enzyme-linked immunosorbent assay (ELISA) analysis***

ELISA analysis was conducted to evaluate the levels of RANKL and OPG during OTM. The analysis was employed using a quantitative sandwich ELISA kit (Cusabio, China). The total amount of each protein was determined using its standard curve. The optical densities were measured at 450 nm using a microplate reader (Bio-Rad Laboratories, USA) and total expressions of RANKL and OPG were presented as pg/ml (pg=picogram/ml= milliliter). Mean values and standard deviations of the mean were calculated for RANKL and OPG levels.

***Statistical analysis***

The results are reported as mean±standard deviation. Intergroup comparisons of OTM rate, RANKL, and OPG levels were performed with an independent sample *t*-test. For all of the tests, differences with values of *P*<0.05 were considered significant. 

## Results

All the experimental procedures were well-tolerated. Furthermore, cocoa administration at the used dosage did not induce any general toxicity including edema and did not affect the body weight of the animals.

Comparison among groups revealed a significant difference between the control groups and those receiving 4.8 g of cocoa. Clinically, Figure 3 shows that the OTM in the treatment group was faster than that of the control group on days 1, 7, and 14 after orthodontic appliance installation. In accordance with clinical observation, the OTM rate measurement of the cocoa group was found to be significantly higher than that of the control group on days 1, 7, and 14 after orthodontic appliance installation (*P*<0.05). Meanwhile, ELISA analysis showed that the OPG level was significantly lower on day 14 after orthodontic appliance installation and that the RANKL level was significantly higher on days 0, 1, and 7 after orthodontic appliance installation in the cocoa group compared with the control group (*P*<0.05, Table 1).

## Discussion

The long duration and complexity of orthodontic treatment may cause many problems. To address this issue, an alternative method has been proposed. In the present study, we evaluated the effect of cocoa on OTM and found it to have a promoting impact on tooth movement in rats. The groups administered with cocoa showed significantly (*P*<0.05) higher RANKL levels, lower OPG levels, and indeed faster OTM than those groups without cocoa. The biochemical mechanisms underlying methylxanthine function can be used to explain our findings. This substance has detrimental effects on bone and has shown potential to induce osteoblast apoptosis by increasing reactive oxygen species production caused by increased cyclic adenosine monophosphate production and raising osteoclast numbers and surface, thereby leading to increased bone resorption (17). 

A correlation exists between osteoblasts and osteoclasts, which mediate osteoclastogenesis. Osteoclast-osteoblast communication contributes to the coupling of bone resorption (18). The process of bone resorption begins with a bond between the reactor activators of nuclear kappa-β ligand (RANKL) produced by osteoblasts in the reactor activator of nuclear kappa-β (RANK) presented in pre-osteoclasts. RANKL subsequently binds to RANK on the surface of osteoclast precursors and recruits the protein adapter, which causes the activation of NF-kB and translocates to the nucleus. NF-kB increases the expression of cFos to trigger osteoclastogenic gene transcription, and finally initiates differentiation of precursor osteoclasts into preosteclast cells. The previous study showed that local RANKL gene transfer significantly accelerates OTM by promoting RANKL expression and improving osteoclastogenesis (19). Conversely, an increase in osteoprotegerin (OPG) level produced by osteoblasts causes a decrease in RANKL level and inhibits OTM (20). OPG is a natural receptor expressed by osteoblasts, which inhibits osteoclast differentiation and activity by binding to the RANKL and blocking RANKL from interacting with RANK (21). The decrease of differentiated osteoblasts is shown by the reduction of OPG expression levels (22). 

In the present study, RANKL expression increased significantly and reached its highest level on day 7 after appliance installation, whereas OPG expression showed a decreasing trend after cocoa administration. These results suggest that the administration of cocoa during active OTM enables a decrease in the levels of OPG and promotes the levels of RANKL. Osteoclast formation and activation are regulated by RANKL through many hormones and cytokines and eventually lead to osteoresorption (23). RANKL signaling ultimately induces expression of cytoplasmic 1 (NFATc1) and osteoclast differentiation-related genes, such as cathepsin K and tartrate-resistant acid phosphatase through nuclear factor of activated T-cells, a key factor in osteoclastogenesis. RANKL also influences osteoclast precursor cells (monocyte-lineage hematopoietic cell) to fuse with one another and become mature multinucleated osteoclast cells, thereby proving the link between RANKL and the activation of osteoclasts (24). 

A previous study has also reported that caffeine can directly decrease bone mineral density by lowering vitamin D receptor protein expression and 1,25(OH)_2_D_3_ serum (25). 1,25(OH)_2_D_3_ is one of the well-known principal regulators of Ca^2+^ homeostasis and is recognized as a regulator of osteoclast-mediated bone resorption and osteoblast-mediated bone formation. When the VDR expression and serum 1,25(OH)_2_D_3_ decrease, the osteoblastic activity may be affected, which may, in turn, contribute to a decrease in bone mineral density (26). This phenomenon corresponds favorably with the research results and explains the link between cocoa administration and acceleration of OTM. 

## Conclusion

The findings of this study prove that cocoa administration contributes to active orthodontic treatment by modulating the rate of tooth movement, inducing osteoclastogenesis, and finally shortening the duration of orthodontic treatment. Further studies are necessary at clinical levels to confirm the efficacy and potency of cocoa in accelerating tooth movement in orthodontic patients.

## References

[1] Andrade JR I, Sousa ABS, Silva GG (2014). New therapeutic modalities to modulate orthodontic tooth movement. Dental Press J Orthod.

[2] Yoshida T, Yamaguchi M, Utsunomiya T, Kato M, Arai Y (2009). Low-energy laser irradiation accelerates the velocity of tooth movement via stimulation of the alveolar bone remodeling. Orthod Craniofac Res.

[3] Yi J, Yan B, Li M, Wang Y, Zheng W (2016). Caffeine may enhance orthodontic tooth movement through increasing osteoclastogenesis induced by periodontal ligament cells under compression. Arch Oral Biol.

[4] Kanzaki H, Chiba M, Arai K, Takahashi I, Haruyama N (2006). Local RANKL gene transfer to the periodontal tissue accelerates orthodontic tooth movement. Gene Ther.

[5] Alhasyimi AA, Pudyani PS, Asmara W, Ana ID (2017). Locally inhibition of orthodontic relapse by injection of carbonated hydroxy apatite-advanced platelet rich fibrin in a rabbit model. Key Eng Mater.

[6] Alhasyimi AA, Pudyani PS, Asmara W, Ana ID (2018). Effect of carbonated hydroxyapatite incorporated advanced platelet rich fibrin intrasulcular injection on the alkaline phosphatase level during orthodontic relapse. AIP Conf Proc.

[7] Iglesias-Linares A, Moreno-Fernandez AM, Yañez-Vico R, Mendoza-Mendoza A, Gonzalez-Moles M (2011). The use of gene therapy vs corticotomy surgery in accelerating orthodontic tooth movement. Orthod Craniofac Res.

[8] Long H, Pyakurela U, Wang Y, Liao L, Zhou Y, Lai W (2013). Interventions for accelerating orthodontic tooth movement: a systematic review. Angle Orthod.

[9] Chen RY, Fu MM, Chih YK, Gau CH, Chiang CY (2011). Effect of cyclosporine-A on orthodontic tooth movement in rats. Orthod Craniofac Res.

[10] Shirazi M, Alimoradi H, Kheirandish Y, Etemad-Moghadam S, Alaeddini M (2014). Pantoprazole, a proton pump inhibitor, increases orthodontic tooth movement in rats. Iran J Basic Med Sci.

[11] Qun Xiao L, Tao Wang H, Lan Li Y, Zeng Q, Zhou E (2015). The effects of dried root aqueous extract of Salvia miltiorrhiza and its major ingredient in acceleration of orthodontic tooth movement in rat. Iran J Basic Med Sci.

[12] Avriliyanti F, Suparwitri S, Alhasyimi AA (2017). Rinsing effect of 60% bay leaf (Syzygium polyanthum wight) aqueous decoction in inhibiting the accumulation of dental plaque during fixed orthodontic treatment. Dent J (Majalah Kedokteran Gigi).

[13] Franco R, Oñatibia-Astibia A, Martínez-Pinilla E (2013). Health benefits of methylxanthines in cacao and chocolate. Nutrients.

[14] Yi J, Zhang L, Yan B, Yang L, Li Y, Zhao Z (2012). Drinking coffee may help accelerate orthodontic tooth movement. Dent Hypotheses.

[15] Shirazi M, Vaziri H, Salari B, Motahhari P, Etemad-Moghadam S, Dehpour AR (2017). The effect of caffeine on orthodontic tooth movement in rats. Iran J Basic Med Sci.

[16] Liu CG, Huang SG, Lin TY, Feng DY, Huang P, Zhang JX (2006). Effect of Erigeron breviscapus on the expression of vascular endothelial growth factor in the periodontal tissues of rabbits during orthodontic tooth movement (In Chinese). Hua Xi Kou Qiang Yi Xue Za Zhi.

[17] Pal S, Khan K, China SP, Mittal M, Porwal K, Shrivastava R (2016). Theophylline, a methylxanthine drug induces osteopenia and alters calciotropic hormones, and prophylactic vitamin D treatment protects against these changes in rats. Toxicol Appl Pharmacol.

[18] Alhasyimi AA, Pudyani PS, Asmara W, Ana ID (2018). Enhancement of post-orthodontic tooth stability by carbonated hydroxyapatite-incorporated advanced platelet-rich fibrin in rabbits. Orthod Craniofac Res.

[19] Kanzaki H, Chiba M, Arai K, Takahashi I, Haruyama N, Nishimura M (2006). Local RANKL gene transfer to the periodontal tissue accelerates orthodontic tooth movement. Gene Ther.

[20] Baloul SS (2016). Osteoclastogenesis and osteogenesis during tooth movement. Front Oral Biol.

[21] d’Apuzzo F, Cappabianca S, Ciavarella D, Monsurrò A, Silvestrini-Biavati A, Perillo L (2013). Biomarkers of periodontal tissue remodeling during orthodontic tooth movement in mice and men: overview and clinical relevance. Scientific World J.

[22] Otero L, García DA, Wilches-Buitrago L (2016). Expression and presence of OPG and RANKL mRNA and protein in human periodontal ligament with orthodontic force. Gene Regul Syst Bio.

[23] Nakano Y, Yamaguchi M, Fujita M, Asano M, Saito K, Kasai K (2011). Expressions of RANKL/RANK and M-CSF/c-fms in root resorption lacunae in rat molar by heavy orthodontic force. Eur J Orthod.

[24] Kohli SS, Kohli VS (2011). Role of RANKL–RANK/osteoprotegerin molecular complex in bone remodeling and its immunopathologic implications. Indian J Endocr Metab.

[25] Rapuri PB, Gallagher JC, Kinyamu HK, Ryschon KL (2001). Caffeine intake increases the rate of bone loss in elderly women and interacts with vitamin D receptor genotypes. Am J Clin Nutr.

[26] Rapuri PB, Gallagher JC, Nawaz Z (2007). Caffeine decreases vitamin D receptor protein expression and 1,25(OH)2D3 stimulated alkaline phosphatase activity in human osteoblast cells. J Steroid Biochem Mol Biol.

